# Lettuce Anaphylaxis in a Florist With Hand Dermatitis and Contact Allergy to the Compositae (*Asteraceae*) Family of Plants

**DOI:** 10.1111/cod.70008

**Published:** 2025-07-30

**Authors:** Lara Obermeyer, Monika Raulf, Irene Mittermann, Martina Aumayr, Christoph Skudlik, Richard Brans

**Affiliations:** ^1^ Institute for Interdisciplinary Dermatologic Prevention and Rehabilitation (iDerm) at the Osnabrück University Osnabrück Germany; ^2^ Department of Dermatology, Environmental Medicine and Health Theory Osnabrück University Osnabrück Germany; ^3^ Institute for Prevention and Occupational Medicine of the German Social Accident Insurance, Institute of the Ruhr‐University Bochum (IPA) Bochum Germany; ^4^ MacroArray Diagnostics GmbH Vienna Austria

**Keywords:** allergic contact dermatitis, anaphylaxis, case report, chicory, compositae, florist, lettuce, occupational, radicchio, sesquiterpene lactones

Lettuce ( *Lactuca sativa* ) belongs to the compositae (*Asteraceae*) family of plants which are known to cause allergic contact dermatitis (ACD) [1]. Rarely, immediate hypersensitivity reactions to lettuce are reported. We here present a case of occupational ACD caused by compositae and sesquiterpene lactones accompanied by a history of lettuce anaphylaxis.

## 1 Case Report

A 49‐year‐old woman of German descent, who was raised in Germany and had worked as a florist for 31 years, presented with dermatitis of her hands and forearms lasting for 6–7 years and showing improvements during periods away from work. She had a history of atopic dermatitis with onset in early childhood affecting various body sites, but no history of hay fever or asthma. She reported several episodes of immediate‐type intolerance reactions after oral intake of butterhead lettuce, romaine lettuce, and radicchio, including a swollen throat, coughing, flush, and abdominal pain. As a result, she had completely avoided lettuce and radicchio in the past year. Physical exertion or intake of non‐steroidal anti‐inflammatory drugs (NSAIDs) as possible cofactors were denied. No other food intolerance was reported. She had not eaten chicory (Belgian endive) in the recent past. Occupational exposure to lettuce or radicchio and work‐related urticaria were denied.

A patch test was performed and read according to the guidelines of the German Contact Dermatitis Research Group (DKG) [2] using the DKG baseline series and the DKG special series for ‘ingredients of topical preparations’, ‘preservatives’, ‘rubber’, ‘plants’, eucalyptus oil 2% pet., and her accelerator‐free protective gloves. The commercial patch test preparations were purchased from SmartPractice Europe (Greven, Germany). Patch tests were removed on day (D) 2 and readings were done on D2, D3, D4, and D7. Positive patch tests were observed on D3 and/or D4 to compositae mix 5% pet. (++), sesquiterpene lactone mix 0.1% pet. (++) and parthenolide 0.1% pet. (++), as well as to thiuram mix 1% pet. (+), tetraethylthiuram disulfide 0.25% pet. (++), 2‐bromo‐2‐nitropropane‐1,3‐diol 0.5% pet. (++), Amerchol L‐101 50% pet. (+), and methyldibromo glutaronitrile 0.2% pet. (+). Based on the patch test results, the history and the exposure assessment, occupational ACD affecting the hands caused by compositae and other plants containing sesquiterpene lactones was diagnosed. No current clinical relevance of the other contact allergies was found.

**FIGURE 1:**
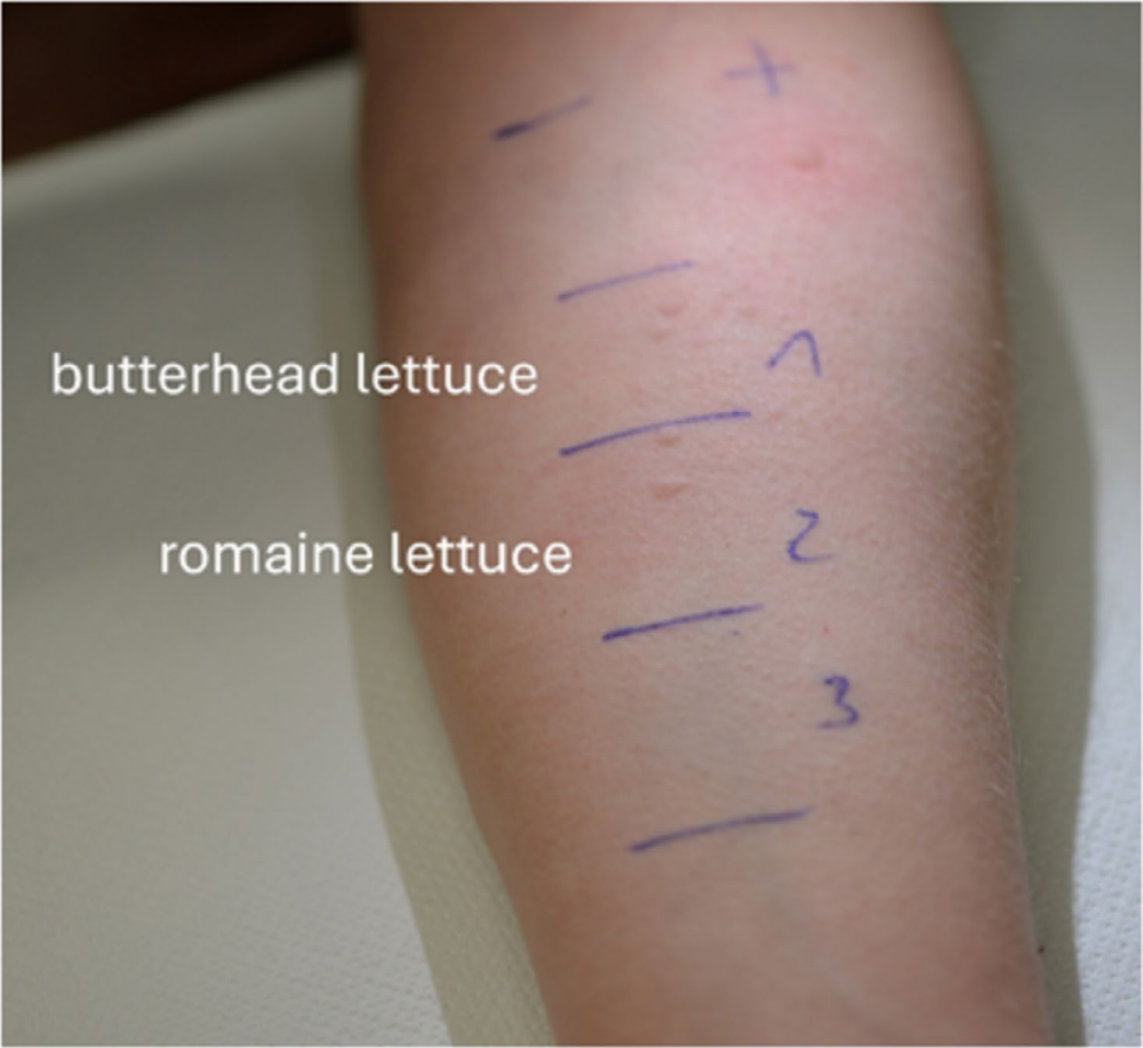
Positive rub test to butterhead lettuce (test area 1) and romaine lettuce (test area 2). Negative rub test to corn starch powder (test area 3) as a negative control. Positive prick test to histamine (+) and negative prick test to saline (−).

Because of the reported immediate‐type reactions after oral intake of lettuce, rub tests with fresh leaves and stems of butterhead lettuce and romaine lettuce were performed on non‐lesional skin of the inner sides of the forearm. The patient developed several wheals (> 3 mm) in both test areas within a few minutes, which disappeared after 40 min (Figure 1) without any delayed or late reactions. Dermographism was ruled out. Rub tests with the same materials in two control subjects were negative. Subsequent prick tests with the patch test preparations of sesquiterpene lactone mix 0.1% pet. and parthenolide 0.1% pet. were negative. Based on the ImmunoCAP assay (Thermo Fisher Scientific Inc., Waltham, MA), the total immunoglobulin (Ig)E serum level was not elevated (52.6 U/mL, reference < 100 U/mL). No specific IgE to recombinant (r)Bet v 1 (birch pollen PR‐10 protein), rBet v 2 (birch pollen profilin), rPhl p 7 (timothy grass pollen polcalcin), rPhl p 12 (timothy grass pollen profillin), rPru p 3 (peach lipid transfer protein), rPla a 1 (plane tree pollen), or lettuce (f215) were detected. In addition, an IgE‐multiplex array (Alex, MacroArray Diagnostics GmbH, Vienna, Austria) covering 295 allergens (including extracts and recombinant allergens of various pollens, plant‐ and animal‐based foods) revealed no detectable IgE sensitizations. However, low levels of IgE (0.31 U_A_/mL) to chicory ( *Cichorium intybus var. foliosum* ) were detected by an additional multiplex array (Fox, MacroArray Diagnostics GmbH, Vienna, Austria). An oral food challenge was not performed.

## 2 Discussion

Compositae (*Asteraceae*) represent a broad plant family. Therefore, positive patch test reactions to Compositae and sesquiterpene lactones, including parthenolide, are very common among florists, frequently causing occupational ACD [3]. Lettuce, including butterhead lettuce and romaine lettuce, belongs to the Compositae family and contains sesquiterpene lactones, such as lactucin, lactucopicrin, and their derivatives [1]. Handling of lettuce could cause occupational ACD in sensitised gardeners, kitchen personnel, or other food handlers [1]. Concomitant immediate hypersensitivity has been reported in a few of these patients, mainly presenting as protein contact dermatitis and/or contact urticaria [1, 4]. Some notice cheilitis and perioral dermatitis as well as localised immediate reactions such as lip swelling or mucosal symptoms after eating lettuce. In these cases, rub or prick‐to‐prick tests with fresh lettuce leaves or stems is recommended [1, 4]. In contrast to the culprit allergens for ACD, proteinaceous allergens are considered causative for the immediate hypersensitivity [1].

Anaphylaxis after consumption of lettuce is very rare. In the present case, no oral food challenge was performed to confirm anaphylaxis to lettuce. The diagnosis was primarily based on the conclusive history of isolated lettuce intolerance and the positive rub test. The non‐specific lipid transfer protein (LTP) *Lactuca sativa* 1 (Lac s 1) has been identified as a culprit allergen in a majority of lettuce anaphylaxis cases [5, 6]. LTPs are pan‐allergens and occur in several plants. LTP‐sensitised individuals, therefore, usually experience symptoms not only to lettuce, but also to a large number of other food and pollen allergens. LTP sensitisations are more common in Mediterranean populations and peach LTP (rPru p 3) is considered the primary sensitiser with a high IgE‐mediated co‐reactivity to Lac s 1 [5, 6]. Lac s 1 as a diagnostic tool was not available. However, since no sensitisation to other LTPs, including rPru p 3 and rPla a 3, was identified, it is highly unlikely that lettuce LTPs were the cause of the lettuce allergy in the present case. However, a rare case of isolated reactivity to Lac s 1 has been reported [5]. A thaumatin‐like protein and an aspartyl protease were additionally identified as IgE‐binding allergens in patients allergic to lettuce [6]. Moreover, profilin was suggested as another lettuce allergen [6]. As these are also pan‐allergens responsible for cross‐reactivity between foods and pollens, it seems similarly unlikely that they are the culprit allergens in this case. More recently, epidermis‐specific EP1‐like glycoprotein and kirola‐like protein were described as novel allergens in farmers with lettuce‐related respiratory allergies [7, 8]. Another option could be cross‐reactivity to chicory ( *cichorium intybus var. foliosum* ) which also belongs to the Compositae family. The patient had low IgE levels to chicory and reported additionally anaphylactic reactions to radicchio, which is a form of leaf chicory. Concomitant immediate‐type allergy to chicory and lettuce based on IgE‐binding to proteins around 42–48 kDa, which is independent from sensitisation to pollens and pan‐allergens, has been described [9, 10].

It is remarkable that the presented case had a medical history of isolated lettuce and radicchio allergy reporting no other food intolerance and that accordingly no specific IgE to other common food and pollen extracts and their recombinant allergens was detected. As a florist, she has been heavily exposed to compositae plants, but not to lettuce or radicchio. We speculate that the occupational exposures to plants did not only induce the delayed‐type sensitisations to compositae and sesquiterpene lactones, but also the immediate‐type sensitisation to an unknown protein which may not only occur in lettuce and radicchio, but also in other compositae plants that she handles at work. Thus, the occupational ACD may have paved the way for the immediate‐type allergy and may have been accompanied by occupational protein contact dermatitis. However, we cannot exclude the independent development of the immediate‐type sensitisation by non‐occupational exposure to lettuce, radicchio or a similar plant facilitated by the impaired skin barrier function related to atopic dermatitis or by oral intake.

## Consent

Written informed consent was obtained to publish the photograph.

## Conflicts of Interest

I. Mittermann and M. Aumayr are employees of MacroArray Diagnostics GbmH. The other authors declare no conflicts of interest.

## Data Availability

Data sharing is not applicable to this article as no new data were created or analyzed in this study.
